# Optimizing hematoma management in axillary osmidrosis surgery: the role of timely drainage and prevention of flap necrosis

**DOI:** 10.1093/jscr/rjae812

**Published:** 2024-12-26

**Authors:** Wen-Tsao Ho

**Affiliations:** Department of Dermatology, Ho Wen Tsao Skin Clinic, No.179, sec 2, Wenhua 3rd Rd., Linkou Dist., New Taipei City 244, Taiwan (R.O.C.)

**Keywords:** osmidrosis surgery, hematoma, drainage, skin necrosis

## Abstract

Hematoma formation is a rare complication following axillary osmidrosis surgery, and its delayed liquefaction can pose significant risks to flap viability, leading to complications such as necrosis. This study examines two cases of postoperative hematomas, highlighting the importance of appropriate drainage management. In both cases, the initial hematomas were evacuated and treated with Penrose drains, but complications arose due to delayed liquefaction. The first case experienced delayed flap necrosis after premature removal of the drain, while the second case benefited from a proactive approach by reintroducing the drain on the sixth postoperative day. This strategy allowed for the prevention of toxic hematoma buildup, improving flap survival. The findings emphasize the need for vigilant postoperative monitoring and drainage management to minimize risks associated with hematoma liquefaction and optimize flap healing. Further research is required to establish standardized protocols for managing postoperative hematomas in axillary osmidrosis surgery.

## Introduction

Axillary osmidrosis surgery is commonly performed to address body odor, but complications such as hematoma formation, although very rare, can pose significant risks to surgical outcomes [1]. If not managed properly, hematomas can lead to severe complications like flap necrosis, ulceration, and impaired wound healing [2]. The timing of Penrose tube placement and removal plays a crucial role in managing these risks. This report discusses two cases of postoperative hematomas following axillary osmidrosis surgery, emphasizing the importance of appropriate drainage timing and the potential harmful effects of hematomas on flap viability.

## Case presentation

### Case 1

**Figure 1:**
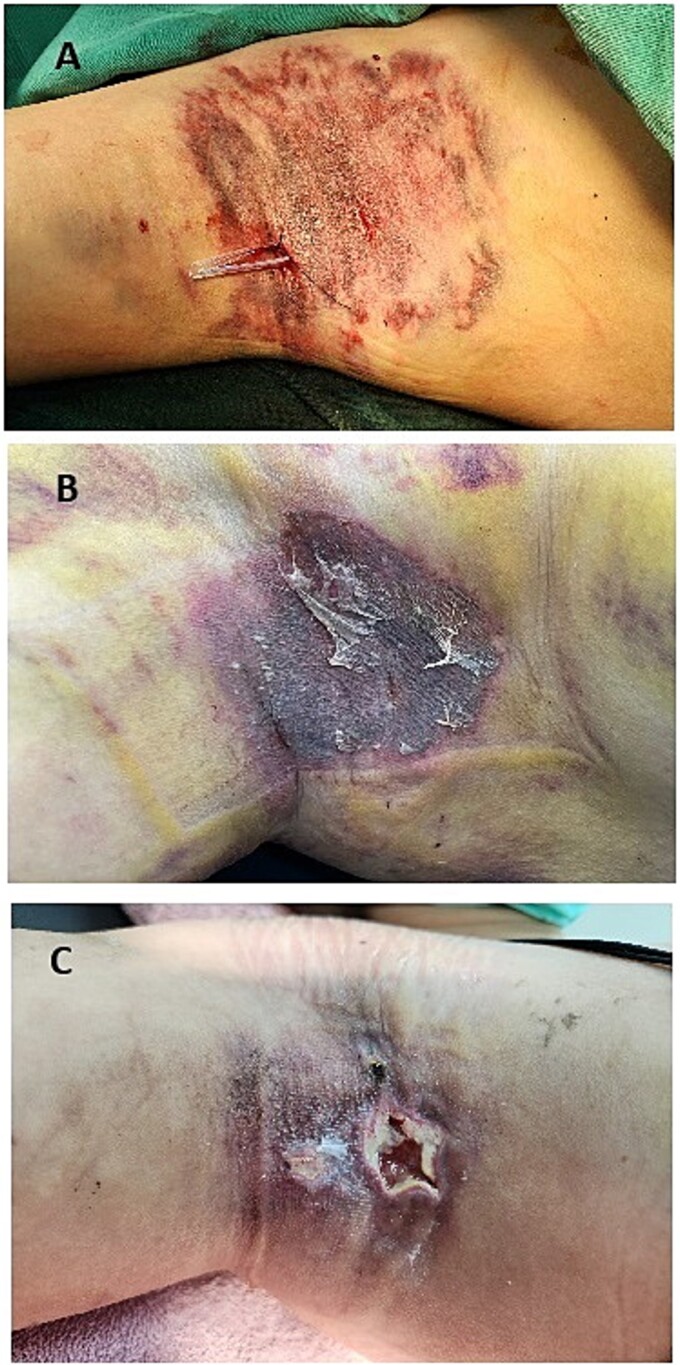
(A) Postoperative image showing the placement of a Penrose drain in the right axilla after hematoma evacuation. The drain is used to facilitate the removal of residual blood and prevent fluid accumulation, promoting proper flap healing. (B) Image taken on the sixth postoperative day, 3 days after the removal of the Penrose drain on the third day. The dressing is clean with no signs of hematoma or active bleeding. The flap appears intact, with no visible complications at this stage. (C) Image taken 3 weeks postoperatively, showing significant necrosis of the flap.

A 28-year-old female underwent surgery for axillary osmidrosis. However, after returning home, she inadvertently applied tension to the surgical site, leading to the formation of a hematoma approximately 10 hours post-operatively. The patient promptly returned to the clinic, where the hematoma was evacuated, bleeding points were controlled with electrocautery, and the area was thoroughly irrigated with normal saline to remove any residual blood. Although the blood clots were effectively cleared, some bruising remained on the skin surface. To facilitate drainage of any remaining blood, a Penrose tube was placed (Fig. 1A), with plans to remove it on the third post-operative day.

The dressing was clean and dry with no signs of active bleeding on the third and sixth days (Fig. 1B), and the flap appeared intact and well-healed from the third to the sixth postoperative day. However, starting from the seventh day, increased exudate was observed on the dressing, progressively worsening each day. This exudate was likely due to liquefied blood leaking from surrounding tissues. The liquefied hematoma became toxic to the flap, eventually leading to necrosis (Fig. 1C).

The management of this case was primarily guided by clinical intuition, recognizing the potential risk of flap compromise despite earlier normal findings. The decision to reintroduce drainage was reactive, responding to visible complications rather than proactively addressing the risk of delayed hematoma liquefaction. The liquefied blood seemed to spread into the surrounding subcutaneous tissue, contributing to necrosis of the flap.

### Case 2

**Figure 2:**
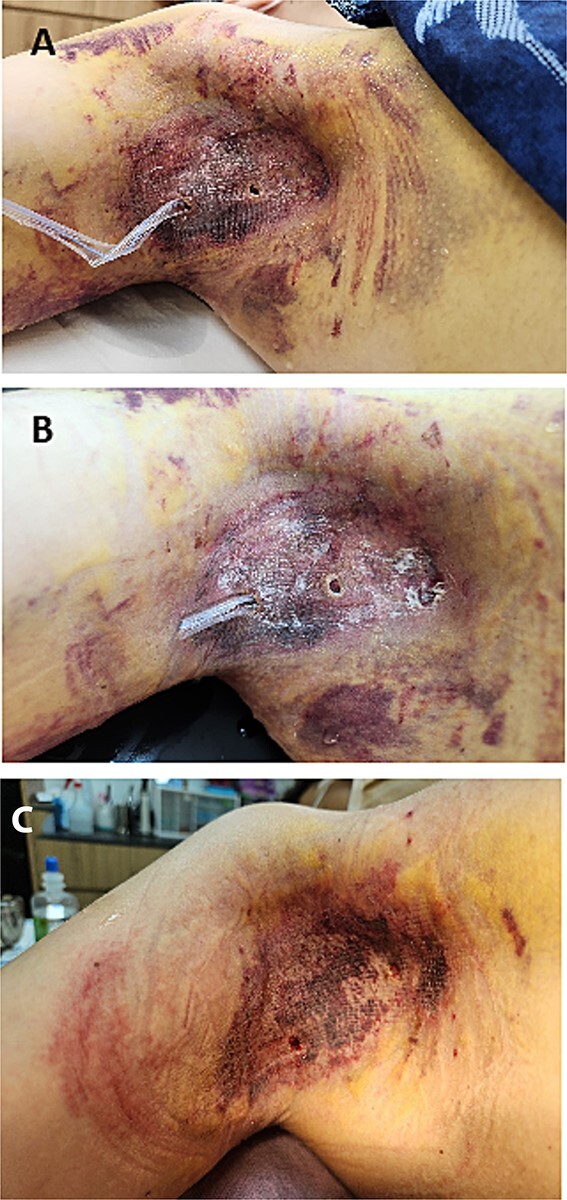
(A) Postoperative image of the right axilla on the third postoperative day, showing the Penrose drain in place with minimal drainage observed. The flap appears intact, with no signs of complications or hematoma. (B) Postoperative image on the sixth postoperative day, where a Penrose drain is reintroduced as a proactive measure to address concerns of delayed hematoma liquefaction. The drainage volume is carefully monitored for any indications of liquefied blood. (C) Postoperative image taken 4 weeks after surgery, showing that the flap has survived without any hematoma formation. The skin appears well-healed, with no signs of complications or necrosis.

A 30-year-old male developed a hematoma after axillary osmidrosis surgery. The hematoma was identified 8 hours postoperatively, likely caused by accidental tension applied to the right axilla during home care activities. Similar to the first case, the patient returned to the clinic, where the hematoma was evacuated, bleeding was controlled, and the area was irrigated. A Penrose tube was placed and removed on the third postoperative day. During this period, minimal drainage was observed, and physical examination revealed no signs of ongoing hematoma, leading to the removal of the drain (Fig. 2A).

However, unlike the first case, a decision was made to reintroduce the Penrose tube on the sixth day, based on the understanding of the potential risks of delayed hematoma liquefaction (Fig. 2B). This proactive measure aimed to address possible complications even in the absence of immediate signs of concern. By the eighth day, the reintroduced drain began to show signs of liquefied blood, confirming the necessity of early intervention. Daily irrigation with 300 cc of saline helped clear the liquefied hematoma, preventing the buildup of toxic by-products that could damage the flap.

This proactive approach was guided by a better understanding of hematoma liquefaction and its impact on flap viability. Reintroducing the Penrose tube early ensured continuous drainage, minimizing the risk of toxic effects on the flap and resulting in better clinical outcomes (Fig. 2C).

## Discussion

These cases highlight the critical role of hematoma management in flap surgery, especially in axillary osmidrosis procedures. Hematomas, even when initially well-managed, can undergo delayed liquefaction, which may jeopardize flap viability due to the release of toxic substances and the promotion of bacterial growth. Proper timing of drain removal and the decision to reintroduce drainage play crucial roles in managing these risks. Understanding hematoma behavior and proactively managing drainage can significantly improve surgical outcomes.

The timing of drainage tube removal is especially critical between the third and seventh post-operative days, as the absence of significant drainage during this period may create a false sense of security. This can lead to premature removal of drainage tubes, despite the potential for delayed liquefaction of hematomas. As demonstrated in these cases, liquefaction of the hematoma can exert toxic effects on the flap even in the absence of immediate visible complications. These delayed effects may not be obvious initially, as the liquefied hematoma can remain hidden beneath the flap and accumulate in dead spaces, making it difficult to detect during routine follow-up examinations.

In both cases, the absence of significant complications initially led to the assumption that healing was progressing as expected. However, complications emerged only days later, illustrating the importance of vigilant monitoring. In the first case, complications were addressed only after visible signs of flap compromise appeared, reflecting a reactive approach. In the second case, the proactive decision to reintroduce a Penrose tube on the sixth day, based on a better understanding of hematoma liquefaction, helped prevent the toxic effects of accumulated hematoma and preserved flap viability, resulting in better clinical outcomes.

Moreover, recent innovations like histotripsy, a technique using high-intensity focused ultrasound, offer a promising approach for managing postoperative hematomas. This technique liquefies large hematomas within 15–20 min, breaking them down with ultrasound waves and making it easier to aspirate the resulting liquid. This method has shown effectiveness in treating large hematomas, and could potentially be applied in axillary osmidrosis surgery. By rapidly liquefying blood clots, histotripsy could prevent complications such as flap necrosis, promote healing, and speed recovery, offering a more efficient and noninvasive option for hematoma management [3].

Effective management of postoperative hematomas is critical to ensuring successful flap healing in axillary osmidrosis surgery. The period between the third and seventh postoperative days is particularly pivotal, as the absence of significant drainage during this time can create a false sense of security, masking the potential risk of delayed hematoma liquefaction. This phenomenon can compromise flap viability if not addressed promptly.

Further research is essential to confirm the efficacy of these strategies and establish standardized, evidence-based protocols for postoperative hematoma management in axillary osmidrosis surgery.
